# Editorial: Introgression Breeding in Cultivated Plants

**DOI:** 10.3389/fpls.2021.764533

**Published:** 2021-09-28

**Authors:** Pietro Gramazio, Jaime Prohens, Laura Toppino, Mariola Plazas

**Affiliations:** ^1^Faculty of Life and Environmental Sciences, University of Tsukuba, Tsukuba, Japan; ^2^Instituto de Biología Molecular y Celular de Plantas, Consejo Superior de Investigaciones Científicas-Universitat Politècnica de València, Valencia, Spain; ^3^Instituto de Conservación y Mejora de la Agrodiversidad Valenciana, Universitat Politècnica de València, Valencia, Spain; ^4^CREA, Council for Agricultural Research and Economics, Research Centre for Genomics and Bioinformatics, Montanaso Lombardo, Italy

**Keywords:** wild relatives, pre-breeding, climate change, diversity, genetic base

The increasing demand for plant products in a climate change scenario is a major challenge for breeders, who are loaded with the pressing need to develop varieties that are more productive, resilient, and efficient in the use of resources (Langridge et al., 2021). Crop wild relatives (CWRs) are a largely unexplored source of genetic variation for many traits, particularly tolerance to biotic and abiotic stresses (Dempewolf et al., 2017), but also for other traits that can contribute to improving the yield, quality, and adaptability of our crops to adverse conditions along with broadening their genetic base (Warschefsky et al., 2014). However, the use of CWRs is not an easy task due to breeding barriers between CWRs and their cultivated relatives that often hamper or, in practice, prevent their use in breeding (Prohens et al., 2017). In addition, CWRs usually display a pattern of traits that are pivotal for survival in the wild, such as physical and chemical defenses, dormancy, or mechanisms for seeds dispersal (easy seed shattering) among others, that turn out undesirable in cultivated species (Meyer and Purugganan, 2013). Despite these difficulties, capturing the useful genetic diversity from CWRs through pre-breeding efforts, combining a broad array of conventional and new molecular techniques, provides new opportunities to develop a new generation of plant materials with improved features introgressed from CWRs (Prohens et al., 2017, Langridge et al., 2021). The urgency to make use of the diversity present in CWRs provided the impetus for this Research Topic. The 14 papers collected here report the development of new introgression materials and strategies, as well as new relevant information, for introgression breeding in a broad range of crops.

Overall, cereals are the primary staple crops in most regions of the world and is one of the crop groups where greater efforts were put into introgression breeding (Dempewolf et al., 2017). Two review papers address the strategies followed in wheat (Hao et al.) and barley (Hernandez et al.). Bread wheat (*Triticum aestivum* L.) is a hexaploid allopolyploid (Feldman and Levin, 2012) with many CWRs exhibiting different ploidy levels in the large *Triticeae* tribe within the *Poaceae* family (Lu and Ellstrand, 2014). Hao et al. presented a strategy based on the exploitation of synthetic hexaploid wheats by double top cross followed by two phases of selection, allowing the development of three new cultivars. This novel strategy could also be extended to other allopolyploid crops where the progenitor species are known. Barley (*Hordeum vulgare* L.) is the fourth most cultivated cereal with two major germplasm groups (two-row vs. six-row) encompassing a large genetic diversity of landraces (Milner et al., 2019). As shown by Hernandez et al., landraces can significantly contribute to barley breeding through introgression into elite material. In this case, the authors introduce past and current efforts for identifying genetic diversity in the barley genepool for improving multiple traits, as well as several case studies for introgression breeding from landraces for stripe and stem rust resistance.

The development of new sets of introgression materials in cereals and their genetic and phenotypic characterization contributes to broadening the genetic base and use in breeding (Lu and Ellstrand, 2014). New lines of wheat with introgression from *Aegilops caudata* L. have been obtained by Grewal et al. after crossing this species with the bread wheat cultivar Paragon, showing the *ph1*/*ph1* mutant genotype, to obtain a set of recombinant introgression lines (ILs) with potential materials carrying the enhanced disease resistance from *A. caudata*. Apart from methods based on sexual crossings, the so-called Exogenous DNA Transfer (EDT) methods allow circumventing hybridization and introgression from CWRs that are not sexually compatible with the crop (Ali et al., 2015; Jiang et al., 2021). One of these methods, the spike-stalk injection method, consisting of the injection of exogenous DNA close to the inflorescence a few days after meiosis (De la Pena et al., 1987), has been exploited by Hu et al. to develop a rice variant with introgressions from *Oryza eichingeri* characterized by higher plant height, panicle length and spikelet number. Using introgression materials developed using CWRs as donors, Bai et al. found resistance to the take-all disease caused by the fungus *Gauemannomyces tritici* in 2Ns/2D substitution line of bread wheat with *Psathyrostachys huashania* Keng. In barley, Hoseinzadeh et al. used a set of ILs with *H. bulbosum* L. to fine map a gene from this latter species conferring resistance to powdery mildew. Finally, Johansson et al. evaluated wheat materials with introgressions from rye, *Leymus* spp. and *Thinopyrum junceiforme* (Á. Löve & D. Löve) Á. Löve for tolerance to several diseases and pests, as well as for quality traits. They found materials with multiple resistances, including resistance to stripe rust, Russian wheat aphid, and Syrian Hessian fly.

Legumes are an important source of protein and oil and can make an effective contribution to sustainable agriculture due to their capacity of symbiotic associations with *Rhizobium* nitrogen-fixing bacteria (Soumare et al., 2020). Pratap et al. reviewed the strategies and achievements made in introgression breeding of legumes by developing different segregating populations for the dissection of traits of interest, mostly for tolerance to biotic and abiotic stresses, existing in the CWRs of eight legumes. Introgression breeding has also resulted in significant improvements in cowpea [*Vigna unguiculata* (L.) Walp.], a multipurpose crop grown in tropical and subtropical regions with high potential for improvement as highlighted by the review of Boukar et al.

Root crops such as potato (*Solanum tuberosum* L.) or sweet potato [*Ipomoea batatas* (L.) Lam.] are important polyploid staple crops for which introgression breeding can make significant contributions for their enhancement and adaptation to climate change (George et al., 2018; Bethke et al., 2019). In fact, potato is one of the crops in which a greater utilization has been made for introgression breeding (Dempewolf et al., 2017), with *Phytophthora infestans* resistance being one of the main targets in the use of CWRs (Su et al., 2020). Rakosy-Tican et al. used somatic hybrids of potato with the resistant species *S. bulbocastanum* Dunal, in which four resistance genes have been identified and cloned, to develop three potato cultivars carrying the resistance genes. Some of the selected clones gave yield and quality similar to the recurrent parent while being resistant. Drought represents a major constrain in some of the major areas of sweet potato cultivation and CWRs carrying tolerance traits to drought have been identified (Nhanala and Yencho, 2020). Guerrero-Zurita et al. subjected 53 accessions of 10 sweet potato CWRs to potential short-memory induction treatment consisting of priming the plants after flowering onset with different water restriction periods. By measuring different ecophysiological indicators they identified *I. triloba* L. and *I. trifida* (Kunth) G. Don as the most promising species for introgression breeding of sweet potato for drought tolerance. In addition, the methodology used can be very efficient for the identification of potential sources of tolerance among CWRs in other crops.

Tomato is one of the crops where most introgression work has been performed, resulting in most of the modern tomato varieties carrying introgressions from several CWRs (Causse et al., 2013). Schouten et al. found that, since the 1960's, the genetic diversity of commercial tomato varieties has continuously increased due to the introgressions from CWRs, mostly aimed at improving resistance to diseases but also fruit quality traits. This article shows how introgression breeding can recover the diversity lost in the different genetic bottlenecks occurring since the domestication of tomato.

Introgression breeding proved also efficient in industrial crops (Dempewolf et al., 2017) where CWRs can contribute to improving biotic and abiotic stress tolerance as well as quality. He et al. genotyped 582 tetraploid cotton (*Gossypium hirsutum* L.) accessions by RAD-seq and found a low diversity in the landraces, while modern cultivars generally contained introgressions from several CWRs which, depending on the introgressed region, contributed to several important traits as also to the genomic differentiation of modern groups.

Introgression breeding programmes may have different levels of complexity depending on the objectives and on the plant materials, genetic diversity, and resources available (Prohens et al., 2017). Therefore, designing an introgression programme is not a trivial task. In this respect, Han et al. have devised a Markov Decision Processes model for optimizing the strategies for the allocation of resources in introgression breeding programmes. The simulations performed show that the model improves the efficiency in the use of resources in the introgression process, making an effective contribution in optimizing the resources available and in consequence maximizing genetic gains.

Overall, the papers from the present Research Topic highlight the importance and efficiency of introgression breeding for broadening the genetic base of our crops and making available to breeders new sources of variation. Despite the effort performed in some major crops (Dempewolf et al., 2017), introgression breeding is still in its infancy in many crops, being challenging in those requiring long generation times, such as fruit trees, or when cross-incompatibility or sterility prevents introgression breeding. In these cases, “new plant breeding techniques” such as cis-genesis and gene editing can contribute to introgression breeding without the need for sexual hybridization (Prohens et al., 2017). In any case, in order to promote introgression breeding among the public and private breeders community, more efforts should be devoted to collecting, conserving and enhancing the CWRs, which are underrepresented in the germplasm collections, as well as to proceed to its extensive high-throughput phenotyping and genotyping. In this way, we are confident that by using the largely unexplored genetic diversity of CWRs and landraces introgression breeding can make an effective contribution to addressing the challenges of improving agricultural productivity and sustainability in a climate change scenario.

## Author Contributions

All authors listed have made a substantial, direct and intellectual contribution to the work, and approved it for publication.

## Conflict of Interest

The authors declare that the research was conducted in the absence of any commercial or financial relationships that could be construed as a potential conflict of interest.

## Publisher's Note

All claims expressed in this article are solely those of the authors and do not necessarily represent those of their affiliated organizations, or those of the publisher, the editors and the reviewers. Any product that may be evaluated in this article, or claim that may be made by its manufacturer, is not guaranteed or endorsed by the publisher.
